# Carbon Dots as a Fluorescent Nanosystem for Crossing the Blood–Brain Barrier with Plausible Application in Neurological Diseases

**DOI:** 10.3390/pharmaceutics17040477

**Published:** 2025-04-06

**Authors:** Catarina Araújo, Raquel O. Rodrigues, Manuel Bañobre-López, Adrián M. T. Silva, Rui S. Ribeiro

**Affiliations:** 1 LSRE-LCM — Laboratory of Separation and Reaction Engineering - Laboratory of Catalysis and Materials, Faculty of Engineering, University of Porto, Rua Dr. Roberto Frias, 4200-465 Porto, Portugal; catarinaisaraujo@gmail.com (C.A.); adrian@fe.up.pt (A.M.T.S.); 2 ALiCE — Associate Laboratory in Chemical Engineering, Faculty of Engineering, University of Porto, Rua Dr. Roberto Frias, 4200-465 Porto, Portugal; 3 Center for MicroElectromechanical Systems (CMEMS-UMinho), University of Minho, Campus de Azurém, 4800-058 Guimarães, Portugal; 4 LABBELS—Associate Laboratory, Braga/Guimarães, Portugal; 5 International Iberian Nanotechnology Laboratory (INL), Av. Mestre José Veiga s/n, 4715-330 Braga, Portugal; manuel.banobre@inl.int

**Keywords:** BBB, bioimaging, dual-emission, nanomedicine, neurological diseases, TEER, transwell permeability

## Abstract

**Background/Objectives**: The development of effective therapies for brain disorders is highly correlated with the ability of drugs or nanosystems to cross the blood–brain barrier (BBB), which has been limited. Recently, carbon dots (CDs) have been receiving attention to be used as BBB-crossing theranostic agents due to their inherent advantages, such as low size, excellent biocompatibility, high quantum yield (QY), tunable fluorescence, high drug loading, and relatively easy synthesis at low cost. The aim of this study was to design CDs with precisely controlled fluorescence properties for advanced bioimaging and an in-depth assessment of BBB permeability. **Methods**: CDs were synthesized using a microwave-assisted approach, optimized through microwaves’ irradiation time, and employing citric acid, urea, and sodium fluoride as precursors. The optimized sample was labeled as NF-CD. **Results**: A comprehensive physicochemical, photoluminescence, and biological characterization revealed the ability of NF-CD to diffuse across a neuromimetic-BBB model, mainly due to their small size (average diameter of 4.0 ± 1.1 nm), exhibiting excitation-dependent fluorescence in the blue and green wavelengths, high biocompatibility and QY, and exceptional photostability. **Conclusions**: Owing to the exceptional fluorescence characteristics and biological compatibility, NF-CD presents promising opportunities in theranostic applications, particularly in brain-targeted bioimaging, nanocarrier-based drug and immunotherapy delivery, early-stage diagnostics, and personalized medicine. NF-CD’s ability to cross the BBB further underscores the relevance of pioneering nanomaterial-based strategies for neurological disorder diagnostics and precision-targeted therapeutic interventions. Overall, this research contributes to the broader field of nanotechnology-driven biomedical advancements, fostering innovations in neurological diagnostics and therapeutic delivery systems.

## 1. Introduction

Neurological disorders, including neurodegenerative Alzheimer’s disease [1] and tumoral gliomas [2], represent a significant global health burden, detrimentally impacting patient quality of life and increasing mortality rates. The complexity of the human brain, consisting of billions of neurons and intricate cellular networks, presents profound challenges in neuroscience and medical research [3].

Delivering neuroprotective or therapeutic agents to specific brain regions is particularly impeded by the highly selective nature of the blood–brain barrier (BBB) [4]. The BBB is a dynamic interface that regulates the molecular exchange between the systemic circulation and the central nervous system, playing a pivotal role in maintaining cerebral homeostasis. The endothelial cells composing the BBB facilitate selective permeability, restricting the passage of potentially neurotoxic compounds, while enabling essential metabolic exchange. Tight junctions and interactions with neurovascular unit constituents, such as astrocytes, further reinforce its restrictive nature. Given these barriers, most small-molecule therapeutics fail to penetrate the BBB, thus the need to develop innovative platforms to study and enhance BBB permeability in neurological diseases [5].

In response to this challenge, carbon dots (CDs) have emerged as promising candidates for biomedical research [6]. These fluorescent quasi-spherical carbon nanomaterials with diameters below 10 nm, occasionally discovered in 2004 [7] and named in 2006 [8], are primarily composed of cores of *sp*^2^-hybridized carbon surrounded by a large amount of oxygen-containing surface functionalities (up to 50 wt.%) [9,10]. High colloidal stability in aqueous suspensions and the high photoluminescence (PL) emission are among the unique properties [11] that have attracted great attention from researchers. The former depends on the number of hydrophilic groups on their surface, while the latter depends on the excitation wavelength, size, and/or functionalization of the CDs [11]. Several other characteristics make CDs promising when compared with traditional semiconductor quantum dots and organic dyes [12], namely their low toxicity, excellent biocompatibility, low cost, high quantum yield (QY), tunable fluorescence, and relatively easy synthesis [6,12,13,14]. In addition, CDs are quickly excreted by the human body and considered environmentally benign [6].

Given the restrictive nature of the BBB and the unique properties of CDs, extensive research has been devoted to evaluating their potential to penetrate this barrier. The physicochemical and biological characteristics of CDs are highly dependent on their synthesis parameters and carbon precursors. The retention of precursor-derived structural motifs and bioactive functionalities [15,16,17] is critical for enhancing BBB permeability. Therefore, the optimization of synthesis methodologies is integral to tailoring the properties of CDs for BBB traversal. Notably, nitrogen doping has been shown to induce red-shifted fluorescence emission, improve the quantum yield, and enhance the biocompatibility of CDs [18], whereas fluoride incorporation has been hypothesized to induce structural and electronic modifications that favor BBB-crossing [19]. In addition to their applications in bioimaging and BBB-crossing, CDs have demonstrated significant potential as therapeutic drug delivery nanosystems in various neurological disorders, including glioblastoma brain tumors [20,21], Parkinson’s disease [22], and Alzheimer’s disease [23], making this nanomaterial an ideal candidate for theranostics.

This study aims to engineer CDs with highly controlled fluorescence properties for advanced bioimaging and an in-depth assessment of BBB permeability. To achieve this, CDs were synthesized using a microwave-assisted approach, employing citric acid, urea, and sodium fluoride as precursors. Microwave synthesis was selected because it provides highly uniform size distributions, fast synthesis, high yield, and low energy consumption [24,25,26].

**Figure 1:**
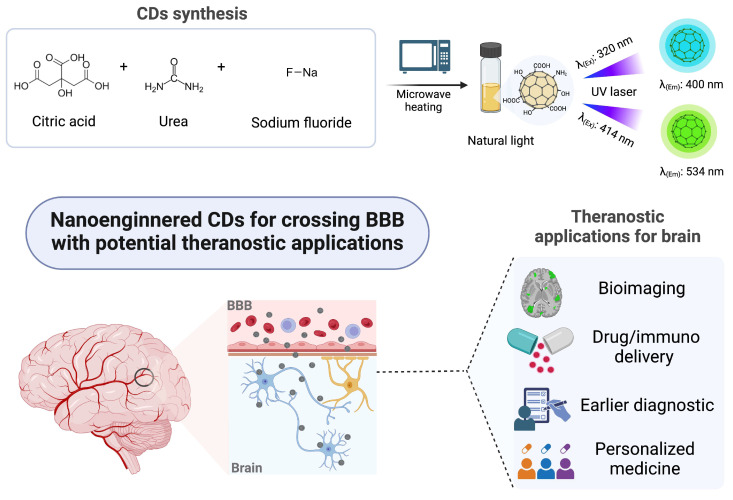
Representation of fluorescent carbon dots (CDs) nanoengineered for crossing the blood–brain barrier (BBB) and their potential as a theranostic nanosystem in neurological diseases.

Based on these goals, we engineered an optimized CD-nanosystem, where its physicochemical and optical properties were systematically analyzed using a suite of characterization techniques. Additionally, CD–BBB interactions were investigated through transwell permeability experiments, viability assays, and fluorescence microscopy. Overall, our findings set this nanoengineered CD-system as a prominent BBB-crossing agent with great potential to be used as a theranostic nanosystem (for both diagnosis and therapy) in neurological diseases, cf. illustrated in Figure 1.

## 2. Materials and Methods

### 2.1. Synthesis of Carbon Dots (CDs)

CDs were synthesized by adapting a microwave-assisted method previously reported elsewhere [27]. Briefly, 0.18 g of citric acid, 0.54 g of urea, and 0.10 g of sodium fluoride were added to 10 mL of ultrapure water. The mixture was dissolved in an ultrasonic bath and then heated in a domestic microwave oven (800 W, Teka, Haiger, Germany) for 3 min. After cooling down to room temperature, the solids were re-dispersed in 10 mL of ultrapure water in an ultrasonic bath for 30 min and purified by dialysis against ultrapure water using a dialysis membrane (Dialysis tubing, Spectrum, molecular weight cutoff: 0.5 kDa) for 24 h. This process resulted in a dark yellow/brownish aqueous suspension containing the sample referred to as NF-CD ready for use. The concentration of each suspension was determined by a dry weight method (Text S1) and then adjusted by adding ultrapure water until the desired value was reached. These suspensions were used to conduct ultraviolet-visible (UV-Vis) and photoluminescence (PL) spectroscopy. NF-CD aqueous suspensions were also freeze-dried to obtain the powder samples needed to carry out additional experiments. Moreover, longer microwave irradiation times were considered during the synthesis procedure, namely 5, 7, 14, 21, and 35 min.

### 2.2. Characterization Techniques

UV-Vis absorbance and photoluminescence (PL) spectra of aqueous suspensions containing each sample of CDs (with a concentration of 13 µg mL^−1^) were collected at room temperature using a JASCO V-560 UV/VIS spectrophotometer (JASCO, Tokyo, Japan) and a JASCO FP-8300 fluorescence spectrometer (JASCO, Tokyo, Japan), respectively. The indirect bandgap (*E*_g_) and transition (*E*_T_) energies were determined from a Tauc plot, with (α*h*υ)^1/2^ as a function of the energy (*h*υ). Raman spectra were recorded at room temperature, with a 20× Zeiss EC Epiplan objective, using a confocal Raman imaging system (Alpha 300, Witec, Ulm, Germany), with 532 nm laser excitation and 1 mW laser power at the focal plane. Three pseudo-Voigt (D, G, and D’) and three Gaussian (D1, D2, and D3) functions were considered in the deconvolution analysis of the Raman spectrum of NF-CD (in the range 1100–1800 cm^−1^) [28]. Fourier transform infrared spectroscopy (FTIR) was performed using a JASCO FTIR-6800 spectrometer (JASCO, Tokyo, Japan) in attenuated total reflection (ATR) mode (256 scans per sample were carried out with a 4 cm^−1^ resolution). The zeta potential was measured on a dynamic light scattering (DLS) apparatus (SZ-100Z, Horiba, Kyoto, Japan), both in ultra-pure water (pH 7.0) and phosphate-buffered saline (PBS, pH 7.4, Corning, New York, NY, USA). The morphology and size of NF-CD were examined using transmission electron microscopy (TEM) with a JEM-2100-HT TEM (JEOL, Tokyo, Japan) operating at 200 kV and equipped with a fast-readout OneView 4k × 4k CCD camera operating at 25 fps (300 fps with 512 × 512 pixels). The specimen for TEM was prepared by dropping an aqueous dispersion of NF-CD onto a copper grid-coated ultra-fine carbon fill (CF300-Cu-UL, Electron Microscopy Science, Hatfield, PA, USA), followed by the evaporation of the solvent.

The photoluminescent quantum yield (QY) was determined by a comparative method using coumarin 153 as a reference. Following IUPAC’s recommendations, this standard was selected because it yields a PL spectrum as identical as possible to that of the CD samples under analysis [29]. Additional details on the QY measurements are provided in Text S2.

### 2.3. Cell Culture Line

The green fluorescent human umbilical vein endothelial cell line (HUVEC-GFP) provided by Innoprot (P20201) was used as cell model to replicate the BBB and perform the cytotoxicity and metabolic assays. The HUVEC-GFP was cultured in a T25 flask supplemented with the EGM Endothelial Cell Growth Medium BulletKit from Lonza Walkersville, and incubated at 5% CO_2_ at 37 °C. At 80% cell confluency, the medium was discarded, the cultures were washed with phosphate-buffered saline (PBS, pH 7.4), and the adherent cells were harvested with 0.25% trypsin for 5 min at 37 °C. The HUVECs were used between passages 2–4.

### 2.4. Cytotoxicity and Metabolic Assays

The HUVEC-GFP was used to assess the cytotoxicity of NF-CD by the AquaBluer assay (Boca Scientific, Dedham, MA, USA), as described by the manufacturer’s protocol. Briefly, the cells were seeded into a 96-well plate at 6 × 10^3^/100 μL/per well, and incubated for 24 h at 37 °C. Then, after removing the cell culture medium, 100 μL of the fresh cell culture medium containing NF-CD ranging from 0 to 500 μg mL^−1^ was added in triplicated wells, and the cells were then incubated for an additional period of 24 h at 37 °C. After this, the cell culture medium was removed and 100 μL of diluted AquaBluer in the cell culture medium (1:100, *v*/*v*) was added to each well, and incubated for 4 h at 37 °C. A plate reader (Synergy, Biotek H1, Winooski, VT, USA) was used to gauge the fluorescence intensity at 540_ex_/590_em_. Non-treated cells were used as a control. The cytotoxicity of NF-CD was calculated based on the read fluorescence intensity (RFI) and expressed as a metabolic activity (%) = (RFI_sample_/RFI_control_) × 100. Additionally, the EC50 was calculated using the four parameter model (OriginPro 2018) to obtain the EC50 values and dose–response curve.

### 2.5. Establishment of Blood–Brain Barrier (BBB) In Vitro Model

The in vitro biomimetic BBB model was established with the HUVEC using transwell systems (cellQART, 1.0 μm PET clean). Briefly, the 12-well cell culture insert transwell were seeded with the HUVEC cells at 1.12 × 10^5^/400 μL/per well-insert, and incubated for 20 days at 37 °C until reaching confluence. Every 2 days, the cell culture medium was exchanged. The trans-epithelial electrical resistance (TEER) of the HUVEC monolayers was measured every 5 days using a Millicell-ERS 3.0 (Millipore, Burlington, MA, USA). The permeability experiments with NF-CD (250 μg mL^−1^) were performed when the TEER reached more than 20 Ω cm^−2^, indicating confluence of the BBB monolayer, as reported elsewhere [30].

### 2.6. Permeability Test in the In Vitro Biomimetic BBB Model

NF-CD at 250 μg mL^−1^ in 400 PBS μL (upper layer) was investigated as the BBB-crossing nanosystem using the in vitro biomimetic BBB model (n = 3). For that, at different time points (30, 60, and 120 min), the fluid in the lower chamber containing NF-CD, which had penetrated the in vitro biomimetic BBB, was collected and transferred to a 96-well plate for measuring the fluorescent signal at 414 nm excitation and 534 nm emission wavelengths (Synergy, Biotek H1 Microplate Reader). At each time point, the lower chamber of the transwell was refilled with fresh PBS to replace the collected sample. A calibration curve was obtained to correlate the permeability concentration of NF-CD with the PL intensity.

## 3. Results and Discussion

### 3.1. Characterization of the Carbon Dots (CDs)

**Figure 2:**
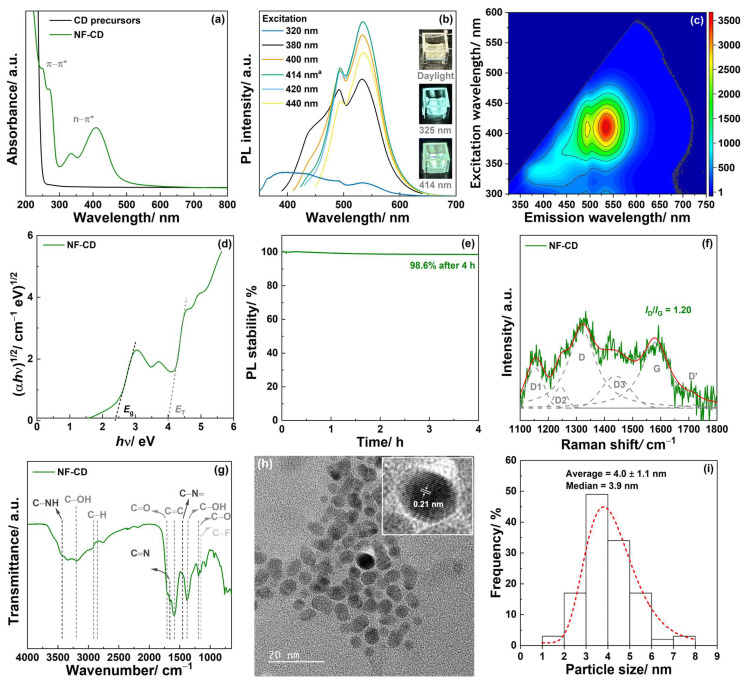
Characterization data. (**a**) UV-Vis absorbance spectra of the precursors used in the synthesis of the CDs and the resulting NF-CD. (**b**) Photoluminescence (PL) spectra, (**c**) contour maps of PL emission, (**d**) Tauc plot, and (**e**) PL stability of NF-CD. (**f**) Deconvoluted Raman spectrum and (**g**) Fourier-transform infrared spectroscopy (FTIR) spectrum of NF-CD. (**h**) Transmission electron microscopy (TEM) micrograph, and (**i**) histogram of particle size distribution (as determined by TEM) of NF-CD. ^a^ in (**b**) denotes the maximum excitation wavelength of NF-CD. Images of aqueous suspensions prepared with NF-CD when subjected to daylight, and 320 and 414 nm radiation are given in the inset of (**b**). The inset of (**h**) is a magnification depicting the lattice spacing of an individual particle of NF-CD. The dashed (red) curve in (**i**) represents a log-normal distribution.

UV-Vis spectroscopy confirmed the conversion of the synthesis precursors upon our microwave-assisted methodology (Figure 2a). As observed, the absorbance spectrum of the aqueous solution containing citric acid, urea, and sodium fluoride (considering the same mass ratios employed in the synthesis procedure) was featureless. On the contrary, the spectrum of NF-CD exhibited absorbance bands that can be attributed to π-π* transitions of the C=C and C=N bonds in the carbon core (at 250 and 270 nm, respectively) and to the n-π* transition of the C=O bonds from different surface groups (at 335 and 410 nm), which are consistent with the presence of CDs composed by the carbon cores surrounded by surface functionalities [27]. The dehydration reactions between the –OH, COOH, and NH_2_ functional groups, followed by carbonization, have been shown the route for the formation of the carbon cores [27].

The PL spectroscopy revealed that the maximum emission is obtained at a wavelength of 534 nm upon excitation at 414 nm (Figure 2b). Accordingly, a light-yellow aqueous suspension of NF-CD (under daylight) emitted the green color when subjected to a 414 nm radiation (inset of Figure 2b). In fact, NF-CD emitted in a broad wavelength range of up to ca. 700 nm (red color region) when excited at 414 nm (Figure 2c). Emissions at lower excitation wavelengths were also observed, yet with lower intensities (Figure 2c). For instance, NF-CD emitted the blue color when subjected to a 320 nm radiation (inset of Figure 2b).

Another important parameter to characterize the PL properties of the CDs is the QY, which is defined as the number of photons emitted relative to the number of photons absorbed [31]. The QY of NF-CD was thus determined as described in Section 2.2 and Text S2. A QY as high as 25.4% was obtained, which may be deemed a good value when compared to the previously reported data on N-doped CDs [13,32] and almost twice as high as the value previously reported for CDs obtained by another microwave-assisted method using citric acid and urea as synthesis precursors (14%) [33]. However, a direct comparison with previous literature data is difficult and may lead to inaccurate conclusions, because the procedures used for the QY determinations are often unclear, and/or standard recommendations, such as those from IUPAC [29], are often overlooked. Interestingly, the QY decreased with the microwave irradiation time considered in the synthesis of our CDs (Figure S1 of the Supplementary Information). Accordingly, only NF-CD was selected for subsequent studies due to its higher QY, namely the bandgap and photostability analyses. Accordingly, the bandgap (*E*_g_) and transition (*E*_T_) energies were determined from the Tauc plot of NF-CD (Figure 2d). An *E*_g_ of 2.38 eV is consistent with the stronger green emission as previously observed by the PL spectroscopy (Figure 2b,c), whereas the blue emission can be ascribed to the *E*_T_ at ca. 4 eV. The intensity of the PL emission yielded by NF-CD after 4 h of continuous excitation at 414 nm was 98.6% of the initial emission (Figure 2e), thus revealing its exceptional photostability. Regarding Raman spectroscopy, the importance of considering the bands other than the three characteristic bands of graphitic materials (i.e., G, D, and D’) has been demonstrated for carbon materials with different dimensionality and structural order [34,35], including CDs [28]. Specifically, including three additional bands (referred to as D1, D2, and D3) during the deconvolution analysis was shown as crucial for the interpretation of the Raman spectra of the CDs [28]. Six contributions were indeed found in the Raman spectrum of NF-CD (Figure 2f and Table S1 of the Supplementary Information). These correspond to the D1 (1153 cm^−1^), D2 (1244 cm^−1^), D (1320 cm^−1^), D3 (1440 cm^−1^), G (1580 cm^−1^), and D’ (1713 cm^−1^) characteristic Raman bands of the CDs [28]. The G, D, and D’ bands result respectively from the stretching of the C–C bond, the presence of disorder in the *sp*^2^-hybridized carbon, and disorder-induced phonon mode due to crystal defects [35,36]. The D1 band results from the presence of the *sp*^2^−*sp*^3^ hybridization at the edges of the CDs, whereas the D2 and D3 bands result from the presence of COOH/C–OH functional groups, and C=O/C–O functional groups, respectively [28]. Accordingly, the presence of structural defects in NF-CD was confirmed by an intensity ratio of the D band relative to the G mode (*I*_D_/*I*_G_) of 1.20, while the ratios *I*_D2_/*I*_G_ and *I*_D3_/*I*_G_ (0.32 and 0.49, respectively; Table S2) suggested an abundance of oxygen surface functional groups. Moreover, the ratio *I*_D1_/*I*_G_ of 0.62 (Table S2) confirms the presence of the *sp*^3^ hybridization on the surface of the carbon cores of NF-CD. FTIR also confirmed the conversion of the synthesis precursors upon our microwave-assisted methodology (Figure S2). A detailed analysis allowed for the confirming of the presence of C=C stretching from non-oxidized domains (band at 1580 cm^−1^) [37,38], as well as the presence of several surface functional groups (Figure 2g). These include mostly oxygen-containing functionalities, namely C–O (1200 cm^−1^), C=O (1710 cm^−1^), and OH (broad band at ca. 3200 cm^−1^ and band at 1370 cm^−1^) [37,38,39,40,41,42], nitrogen-containing functionalities, namely C–N= (1450 cm^−1^), C=N (1665 cm^−1^), and NH (3425 cm^−1^) [27,41,42]. Nevertheless, the presence of C–F bounds was also suggested by a band at 1150 cm^−1^ [43]. This is an important feature, as the presence of fluoride in nanosystems has been shown to increase their ability for BBB-crossing, either by inducing structural and electronic modifications [19], or by increasing the hydrophobicity and reducing the surface energy [44].

A mean zeta potential of −36.8 mV (Figure S3a, ultrapure water) confirmed the excellent colloidal stability of NF-CD, which can be attributed mostly to the presence of hydrophilic functional groups and the small size of NF-CD (Figure 2h). Additionally, the zeta potential was measured in PBS at a pH of 7.4 to assess the colloidal stability of NF-CD in the presence of salts and under physiological pH, representing biological conditions. As expected, a decrease in the negative zeta potential was observed, attributed to the compression of the electrical double layer by the salts. Nevertheless, the mean zeta potential of −12.4 mV still indicates good colloidal stability, supporting the potential use of this nanomaterial in biomedical applications (Figure S3b, PBS). In fact, a narrow particle size distribution was found (Figure 2i), corresponding to an average diameter of 4.0 ± 1.1 nm. Transmission electron microscopy (TEM) also allowed for the estimating of the lattice spacing of NF-CD (inset of Figure 2h). The obtained value (0.21 nm) is consistent with the presence of *sp*^3^-hybridized carbon at the edges of the CDs, as previously reported for different CDs [13].

The above results confirm that our microwave-assisted method is able to yield CDs with an average diameter of 4.0 ± 1.1 nm and dual-emission behavior (blue or green color depending on the excitation wavelength).

### 3.2. Cytotoxicity and Metabolic Activity of NF-CD in BBB Model Cells

**Figure 3:**
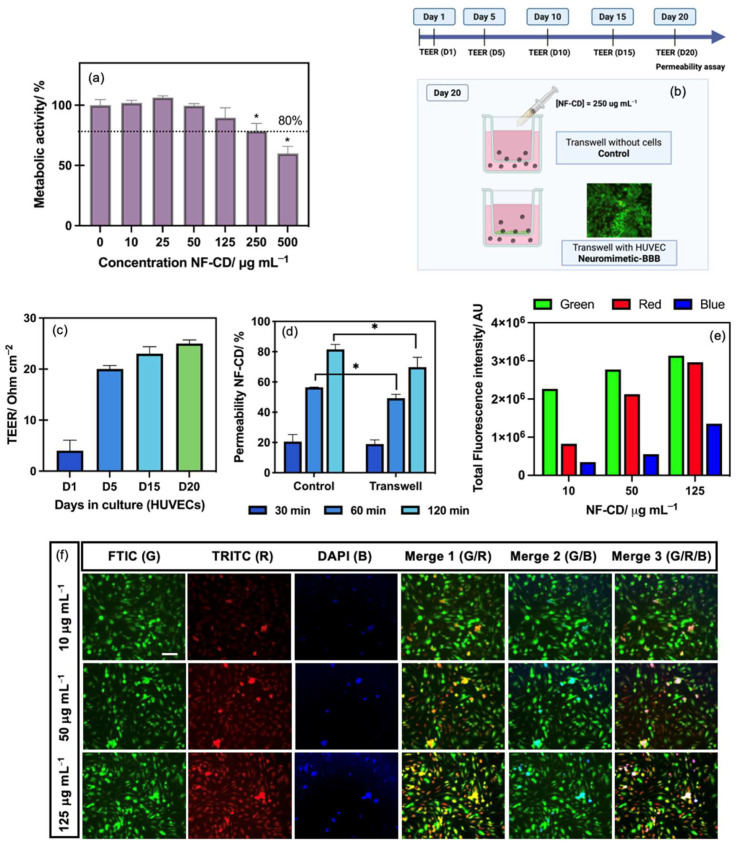
Biological characterization data. (**a**) Determination of the cytotoxicity of NF-CDs, ranging from 0 to 500 μg mL^−1^ and expressed as metabolic activity using the HUVECs. A two-tailed paired *t*-test was used compared with the control; bars represent the mean ± s.d. (* *p* < 0.05). (**b**) Illustrative representation of the establishment of the BBB in vitro model using transwell systems. (**c**) Trans-epithelial electrical resistance (TEER) monitoring of the neuromimetic-BBB along 20 days of culture. (**d**) Permeability assessment of NF-CD, loaded at 250 μg mL^−1^, at different time points (30, 60, 120 min) in the neuromimetic-BBB and control transwell systems without cells. A two-tailed unpaired *t*-test was used; bars represent the mean ± s.d. (* *p* < 0.05). (**e**) Total fluorescence intensity of NF-CD cultured with the HUVEC at different concentrations (10, 50, and 125 μg mL^−1^), obtained with green (FTIC), red (TRITC), and blue (DAPI) filters. (**f**) Bioimaging of the HUVEC cultured with NF-CD at different concentrations (10, 50, and 125 μg mL^−1^) for 24 h with green (FTIC), red (TRITC), and blue (DAPI) filters, as well as their merging fluorescent images (Merge 1, green and red, Merge 2, green and blue, and Merge 3, green, red, and blue). Scale bar = 100 μm.

In addition to a high QY and excellent photostability, low cytotoxicity is another essential requirement for CDs in biomedical applications, including for bioimaging and therapeutic cargo-nanovector. To determine NF-CD’s cytotoxicity, HUVEC (a typical vascular endothelial cell line that models BBB) was cultured with a series of concentrations, ranging from 0 to 500 μg mL^−1^, exposed for 24 h, and their metabolic activity was assessed by the AquaBluer assay. The results showed that NF-CD has a low cytotoxicity over the HUVEC, expressing high metabolic activity (≥80%) until 250 μg mL^−1^ when compared with the control cells (Figure 3a). Based on these data, the EC50 and dose–response curve was calculated to be at 671 μg mL^−1^, which expresses the good biocompatibility of these CDs to be used in biomedical applications.

### 3.3. BBB Permeability Ability of NF-CD

The brain has evolved with an extra-protective barrier of vascular endothelial cells (the so-called BBB) that prevents the central nervous system from toxins and pathogens in the blood [45]. The microcapillary endothelium is distinguished by the presence of tight junctions, the absence of fenestrations, and a minimal number of pinocytotic vesicles [46]. This natural protective barrier is responsible for excluding over 98% of small-molecule drugs (below 500 Da) and blocks all the macromolecular therapeutics from accessing the brain [46]. Nevertheless, the tight junctions in the BBB feature gaps of 4–6 nm, indicating that nanoengineered systems smaller than 6 nm may pass through these gaps [47]. Given that the particle size of NF-CD is approximately 4.0 nm, its potential to traverse the BBB was assessed using a BBB-biomimetic model. This biomodel emulates the anatomical structure of brain capillaries using the HUVECs seeded in transwell systems (Figure 3b). To guarantee the transendothelial resistance and monolayer integrity, as found in vivo, the TEER was measured for 20 days until a value above 20 Ω cm^−2^ was reached (Figure 3c). The average TEER values between 14–34 Ω cm^−2^ are typically reported for the HUVEC monolayers cultured using transwell systems [30]. The ability of NF-CD to penetrate the BBB was assessed by tracking the fluorescence intensity of the CDs that accumulated in the lower chamber of the biomimetic BBB transwell system. As illustrated in Figure 3d, NF-CD successfully crossed the BBB in a time-dependent manner, a characteristic attributed to their small size. In comparison with the control transwell systems (without cells), NF-CD achieved 70% of permeability in the fully developed BBB versus 81% in the control, in just 2 h. Overall, the BBB-crossing capacity highlights its potential to be used in biomedical applications for neurological diseases, such as bioimaging and potential therapeutic cargo-nanovector. Additionally, the bioimaging capability was evaluated through fluorescence microscopy as an optical nanoprobe using different fluorescent channels [i.e., green (FTIC), blue (DAPI), and red (TRITC)]. The results showed that besides the green and blue emissions, NF-CD also emits fluorescence in the red region (Figure 3e,f). These results agree with the PL emission maps, where emissions at wavelengths above 570 nm (TRITC emission wavelengths) were observed (Figure 2c). By shielding the wavelengths corresponding to the green and blue regions, the bandpass filters of the fluorescence microscope allowed for observing a clear and strong signal at red wavelengths. This feature not only allowed for distinguishing the PL emission of the HUVEC cells (in the green region) from that of NF-CD (in the red region), but also allowed for confirming the ability of NF-CD to be internalized by those cells (as shown by the extensive overlapping between the green and red fluorescent signals observed when merging the two channels, cf. Figure 3f). Also, an increase in the PL intensity with the NF-CDs concentration was observed.

## 4. Conclusions

Our microwave-assisted method enabled the synthesis of CDs (referred to as NF-CD), with an average particle size of 4.0 ± 1.1 nm and an abundance of oxygen- and nitrogen-containing surface functional groups. Moreover, a successful incorporation of fluoride was suggested by FTIR. As a result of these unique properties, NF-CD revealed a QY of 25.4%, dual-emission behavior (blue or green color, depending on the excitation wavelength), and excellent colloidal and photostability, as respectively confirmed by a negative Zeta potential and the ability to maintain 98.6% of the initial PL emission after 4 h of continuous excitation.

The unique features above, the demonstrated NF-CD’s low cytotoxicity toward a typical vascular endothelial cell line that models the BBB, and the ability to penetrate and cross a BBB-biomimetic model make it a highly promising nanosystem for brain-targeted bioimaging, nanocarrier-based drug and immunotherapy delivery, early-stage diagnostics, and personalized medicine.

## Data Availability

The original contributions presented in this study are included in the article/supplementary material. Further inquiries can be directed to the corresponding authors.

## Supplementary Materials

The following supporting information can be downloaded at https://www.mdpi.com/article/10.3390/pharmaceutics17040477/s1, Text S1: Determination of CDs’ concentration in suspension; Text S2: Detailed description of the photoluminescent quantum yield (QY) measurements; Figure S1. Quantum yield (QY) as a function of the microwave irradiation time considered in the synthesis of the CDs; Figure S2. Fourier-transform infrared spectroscopy (FTIR) spectra of the synthesis precursors and the resulting NF-CD; Figure S3. Zeta potential spectra of NF-CD. (a) ultrapure water (pH 7.0). (b) Phosphate-buffered saline (PBS, pH 7.4); Table S1: Results obtained from the deconvolution of the Raman spectrum of NF-CD; Table S2: Intensity ratios obtained from the deconvolution of the Raman spectrum of NF-CD. References [48,49] are cited in the supplementary materials.

## Abbreviations

The following abbreviations are used in this manuscript:

ATR	Attenuated total reflection
BBB	Blood–brain barrier
CDs	Carbon dots
DLS	Dynamic light scattering
FTIR	Fourier transform infrared spectroscopy
*I*_D_	Intensity of the D band in the Raman spectrum
*I*_D2_	Intensity of the D2 band in the Raman spectrum
*I*_D3_	Intensity of the D3 band in the Raman spectrum
*I*_G_	Intensity of the G band in the Raman spectrum
PL	Photoluminescence
QY	Quantum yield
TEM	Transmission electron microscopy
UV-Vis	Ultraviolet-visible
