# Supplementary material for: Carbon Dots as a Fluorescent Nanosystem for Crossing the Blood–Brain Barrier with Plausible Application in Neurological Diseases
Source: Pharmaceutics. 2025 Apr 6;17(4):477. doi: 10.3390/pharmaceutics17040477 (PMC12030320; doi:10.3390/pharmaceutics17040477)
Supplement: Supplementary file 1 [file pharmaceutics-17-00477-s001.zip › pharmaceutics-3549114-supplementary.pdf]

Supplementary Information

Carbon Dots as a Fluorescent Nanosystem for Crossing the Blood–Brain Barrier with Plausible Application in Neurological Diseases

Catarina Araújo [1,2], Raquel O. Rodrigues [3,4,5,*] Manuel Bañobre-López [5], Adrián M. T. Silva [1,2] and Rui S. Ribeiro [1,2,*]

[1] *LSRE-LCM—Laboratory of Separation and Reaction Engineering, Laboratory of Catalysis and Materials, Faculty of Engineering, University of Porto, Rua Dr. Roberto Frias, 4200-465 Porto, Portugal; catarinaisaraujo@gmail.com (C.A.); adrian@fe.up.pt (A.M.T.S.)*
[2] *ALiCE—Associate Laboratory in Chemical Engineering, Faculty of Engineering, University of Porto, Rua Dr. Roberto Frias, 4200-465 Porto, Portugal*
[3] *Center for MicroElectromechanical Systems (CMEMS-UMinho), University of Minho, Campus de Azurém, 4800-058 Guimarães, Portugal*
[4] *LABBELS—Associate Laboratory, Braga/Guimarães, Portugal*
[5] *International Iberian Nanotechnology Laboratory (INL), Av. Mestre José Veiga s/n, 4715-330 Braga, Portugal; manuel.banobre@inl.int*

* Correspondence: raquel.rodrigues@dei.uminho.pt (R.O.R) and rsribeiro@fe.up.pt (R.S.R.)

Contents

Supplementary Discussion

Text S1. Determination of CDs' concentration in suspension. **Page 3**.

Text S2. Detailed description of photoluminescent quantum yield (QY) measurements. **Page 3**.

Supplementary Figure

Figure S1. Quantum yield (QY) as a function of the microwave irradiation time considered in the synthesis of the CDs. **Page 3.**

Figure S2. Fourier-transform infrared spectroscopy (FTIR) spectra of the synthesis precursors and the resulting NF-CD. **Page 4.**

Figure S3. Zeta potential spectra of NF-CD. (a) ultrapure water (pH 7.0). (b) Phosphate-buffered saline (PBS, pH 7.4). **Page 4**.

Supplementary Tables

Table S1. Results obtained from the deconvolution of the Raman spectrum of CD_3. **Page 5**.

Table S2. Intensity ratios obtained from the deconvolution of the Raman spectrum of CD_3. **Page 5**.

References. Page 6.

Text S1. Determination of CDs' concentration in suspension

The concentration of each CD sample in the purified suspensions was determined by drying off the water at 100 ºC. For that purpose, 2 mL aliquots were added to dried vials (in triplicate), kept at 100 ºC for 24 h, and weighted. The concentration in the testing suspension was then obtained by the difference between the mass of the CD-containing and empty vials.

Text S2. Detailed description of photoluminescent quantum yield (QY) measurements

The QY of each sample (QY_{Sample}) was obtained as described in Eq. S1 [48], where $QY_{Coumarin\ 153}$ is the QY of coumarin 153 (0.12) [49]; m_{Sample} and $m_{Coumarin\ 153}$ are the slopes of the lines obtained from the plots of integrated PL intensity at an excitation wavelength of 430 nm *vs*. absorbance of the sample and standard, respectively; and η_{Sample} and $\eta_{Coumarin\ 153}$ are the refractive index (η) of the solvent used to prepare the sample suspension and the standard solution, respectively (in this case, water for both; with η = 1.33). 5 solutions with absorbance in the range of 0.01 – 0.1 were analyzed for each sample and the standard. To correct for possible oxygen quenching of fluorescence, samples/standards were purged with nitrogen for 2 minutes immediately before the PL measurements.

$$QY_{Sample} = QY_{Coumarin\ 153} \times \left(\frac{m_{Sample}}{m_{Coumarin\ 153}}\right) \times \left(\frac{{\eta_{Sample}}^2}{{\eta_{Coumarin\ 153}}^2}\right) \qquad \textbf{(S1)}$$

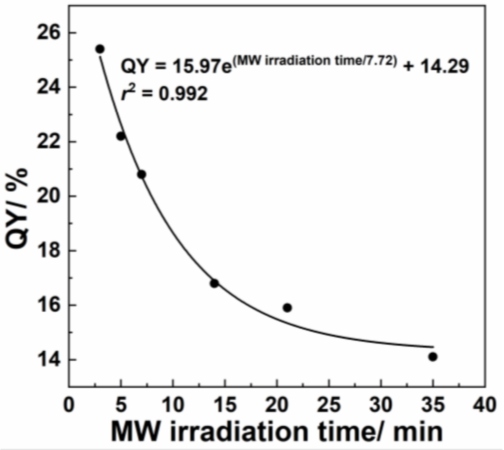

Figure S1. Quantum yield (QY) as a function of the microwave irradiation time considered in the synthesis of the CDs.

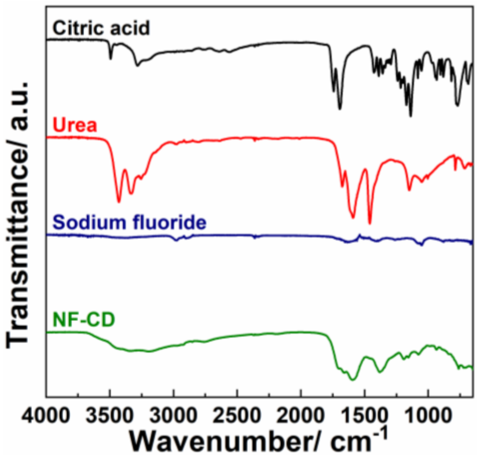

Figure S2. Fourier-transform infrared spectroscopy (FTIR) spectra of the synthesis precursors and the resulting NF-CD.

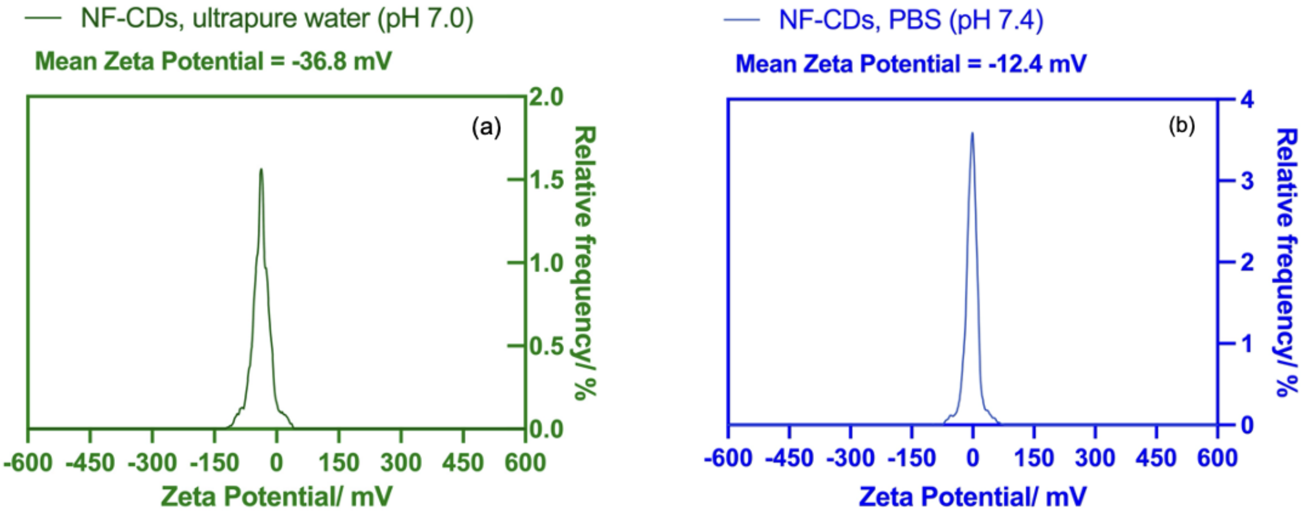

Figure S3. Zeta potential spectra of NF-CD. (a) ultrapure water (pH 7.0). (b) Phosphate-buffered saline (PBS, pH 7.4).

Table S1. Results obtained from the deconvolution of the Raman spectrum of CD_3. X_c, I, and W represent the peak center, peak intensity, and width of the peak at half-height, respectively.

D1 (Gauss)			D2 (Gauss)			D (Psd-Voigt)			D3 (Gauss)			G (Psd-Voigt)			D' (Psd-Voigt)		
X_c/ cm^{-1}	I/ %	W/ cm^{-1}	X_c/ cm^{-1}	I/ %	W/ cm^{-1}	X_c/ cm^{-1}	I/ %	W/ cm^{-1}	X_c/ cm^{-1}	I/ %	W/ cm^{-1}	X_c/ cm^{-1}	I/ %	W/ cm^{-1}	X_c/ cm^{-1}	I/ %	W/ cm^{-1}
1153	51.4	70	1244	27.1	50	1320	100	120	1440	40.7	120	1580	83.3	120	1713	11.3	50

Table S2. Intensity ratios obtained from the deconvolution of the Raman spectrum of CD_3. Intensity ratios of the D1, D2, D, D3, and D' bands relative to the G mode.

I_{D1}/ I_G	I_{D2}/ I_G	I_D/ I_G	I_{D3}/ I_G	$I_{D'}$/ I_G
0.62	0.32	1.20	0.49	0.14

References

48. Kandi, D.; Mansingh, S.; Behera, A.; Parida, K. Calculation of relative fluorescence quantum yield and Urbach energy of colloidal CdS QDs in various easily accessible solvents. *J. Lumin.* **2021**, 231, 117792. https://doi.org/10.1016/j.jlumin.2020.117792.

49. Jones II, G.; Jackson, W.R.; Choi, C.Y.; Bergmark, W.R. Solvent effects on emission yield and lifetime for coumarin laser dyes. Requirements for a rotatory decay mechanism, J*. Phys. Chem.* **1985**, 89, 294-300. https://doi.org/10.1021/j100248a024.
